# Radon Exposure, IL-6 Promoter Variants, and Lung Squamous Cell Carcinoma in Former Uranium Miners

**DOI:** 10.1289/ehp.1409437

**Published:** 2015-09-15

**Authors:** Shuguang Leng, Cynthia L. Thomas, Amanda M. Snider, Maria A. Picchi, Wenshu Chen, Derall G. Willis, Teara G. Carr, Jacek Krzeminski, Dhimant Desai, Amin Shantu, Yong Lin, Marty R. Jacobson, Steven A. Belinsky

**Affiliations:** 1 Lung Cancer Program, Lovelace Respiratory Research Institute, Albuquerque, New Mexico, USA; 2 Saccomanno Research Institute, St. Mary’s Medical Center, Grand Junction, Colorado, USA; 3 Department of Pharmacology, Penn State College of Medicine, Hershey, Pennsylvania, USA

## Abstract

**Background:**

High radon exposure is a risk factor for squamous cell carcinoma, a major lung cancer histology observed in former uranium miners. Radon exposure can cause oxidative stress, leading to pulmonary inflammation. Interleukin-6 (IL-6) is a pro-carcinogenic inflammatory cytokine that plays a pivotal role in lung cancer development.

**Objectives:**

We assessed whether single nucleotide polymorphisms (SNPs) in the IL6 promoter are associated with lung cancer in former uranium miners with high occupational exposure to radon gas.

**Methods:**

Genetic associations were assessed in a case–control study of former uranium miners (242 cases and 336 controls). A replication study was performed using data from the Gene Environment Association Studies (GENEVA) Genome Wide Association Study (GWAS) of Lung Cancer and Smoking. Functional relevance of the SNPs was characterized using in vitro approaches.

**Results:**

We found that rs1800797 was associated with squamous cell carcinoma in miners and with a shorter time between the midpoint of the period of substantial exposure and diagnosis among the cases. Furthermore, rs1800797 was also associated with lung cancer among never smokers in the GENEVA dataset. Functional studies identified that the risk allele was associated with increased basal IL-6 mRNA level and greater promoter activity. Furthermore, fibroblasts with the risk allele showed greater induction of IL-6 secretion by hydrogen peroxide or benzo[a]pyrene diolepoxide treatments.

**Conclusions:**

An IL6 promoter variant was associated with lung cancer in uranium miners and never smokers in two external study populations. The associations are strongly supported by the functional relevance that the IL6 promoter SNP affects basal expression and carcinogen-induced IL-6 secretion.

**Citation:**

Leng S, Thomas CL, Snider AM, Picchi MA, Chen W, Willis DG, Carr TG, Krzeminski J, Desai D, Shantu A, Lin Y, Jacobson MR, Belinsky SA. 2016. Radon exposure, IL-6 promoter variants, and lung squamous cell carcinoma in former uranium miners. Environ Health Perspect 124:445–451; http://dx.doi.org/10.1289/ehp.1409437

## Introduction

Radon is an inert gas released during the decay of radium-226. Radon gas is ubiquitous in indoor and outdoor air and contaminates many underground mines (Sethi et al. 2012). Cohort studies of underground miners have established a strong association between high levels of radon exposure and increased risk for lung cancer (Archer 1988; Archer et al. 2004; Gilliland et al. 2000). Moreover, combined exposure to radon and tobacco smoke through uranium mining may further increase lung cancer risk (Archer 1988). Lung squamous cell carcinoma is the predominant histological type of lung cancer observed in former uranium miners, and it is likely driven by the localization of radon within the upper airways owing to its binding to silica and diesel particles inhaled by the miners (Saccomanno et al. 1996; Samet 1989; Sethi et al. 2012). An association between residential radon exposure and lung cancer risk was also identified in seven North American case–control studies (Krewski et al. 2006).

The high–linear energy transfer alpha particles emitted by radon and radon daughters can directly attack genomic DNA and cause mainly double-strand breaks in DNA (Prise et al. 2001; Sethi et al. 2012). In addition, overproduction of reactive oxygen species in the lungs caused by persistent radon exposure may cause oxidative stress, leading to pulmonary inflammation, tissue damage, and eventually to chronic lung diseases such as chronic obstructive pulmonary disease (COPD), pulmonary fibrosis, and lung cancer (Archer et al. 1998; Iyer et al. 2000; Mapel et al. 1997; Narayanan et al. 1997; Rosanna and Salvatore 2012; Schubauer-Berigan et al. 2009). Strong associations between pulmonary inflammation as manifested in COPD and/or chronic mucous hypersecretion and risk for subsequent lung cancer incidence support the premise that persistent inflammation is involved in the etiology of lung cancer (Brenner et al. 2012; Wilson et al. 2008). Among the cytokines and chemokines produced by persistent pulmonary inflammation, interleukin-6 (IL-6) plays a pivotal role in promoting cancer development as shown in studies using *in vitro* and *in vivo* models of lung carcinogenesis (Chen et al. 2012; Dougan et al. 2011; Gao et al. 2007; Ochoa et al. 2011; Qi et al. 2014).

*IL6* has a promoter with several single nucleotide polymorphisms (SNPs) that show large differences in minor allele frequency (MAF) across major ethnic populations. Increased induction of *IL6* promoter activity by norepinephrine, lipopolysaccharide, and IL-1 in an *in vitro* plasmid construct carrying the *G* allele of rs1800795 has been identified (Cole et al. 2010; Fishman et al. 1998). Gel shift assays confirmed the exclusive binding of GATA1 to the sequence containing the rs1800795 *G* allele following norepinephrine induction (Cole et al. 2010). However, results from population studies assessing the association between rs1800795 and plasma levels of IL-6 as an indicator for systematic inflammation were inconsistent (Fishman et al. 1998; He et al. 2009; Ljungman et al. 2009a, 2009b; Sousa et al. 2012; Zakharyan et al. 2012).

The present study assessed the association between *IL6* promoter SNPs and squamous cell carcinoma in uranium miners. Generalization to populations with residential radon exposure was examined using the Gene Environment Association Studies (GENEVA) Genome Wide Association Study (GWAS) of Lung Cancer and Smoking. The functional relevance of significant variants was also assessed *in vitro* using multiple cell types.

## Methods

*Former uranium miners.* A cumulative incidence case–control study was conducted in the Saccomanno Uranium Miner cohort of male former uranium miners (*n* = 17,000) who worked underground at the Colorado plateau and participated in sputum cytology screening for lung cancer detection between 1957 and 2002 (Saccomanno et al. 1996). A pool of confirmed deceased former uranium miners (360 squamous cell carcinoma cases and 810 lifetime lung cancer–free miners) with available data for essential variables including age and smoking history at sputum collection, working level month (WLM), age at death, age at lung cancer diagnosis (for cases), and survival after lung cancer diagnosis (for cases) were used in the present study. Squamous cell carcinoma cases were identified from the St. Mary’s Hospital Cancer Registry and the St. Mary’s Saccomanno Research Institute Cancer Research Database. Because 94% of the miners (*n* = 1,100) were Caucasian, the study was restricted to this ethnic group to minimize bias owing to ancestry differences. The sputum specimens proximal to cancer diagnosis (cases) or the last follow-up examination (controls) were used for DNA isolation. Study participants for whom DNA samples could not be recovered from sputum cytology slides based on amplification of a 180-bp DNA fragment in the human *KRAS* gene were excluded from the present study. A total of 267 cases and 383 controls were included for the genotyping study. For the miners selected as the controls, cumulative radon exposure at work, expressed as WLM, and age at death were similar to those reported for other lifetime lung cancer–free uranium miners who were born during 1904–1933 [WLM, 0.8 vs. 0.7 kWLMs; age at death, 68.7 vs. 68.5 years (Leng S., unpublished data)]. No personal identifiers accompanied the transfer of material from the St. Mary’s Saccomanno Research Institute to Lovelace Respiratory Research Institute. The present study was conducted under an Institutional Review Board–approved protocol that was exempt from informed consent requirements based on the Department of Health and Human Services regulations under 45CFR46.102(f), which defines a human subject as a living individual.

*Cumulative radon exposure.* WLM, a time-integrated measure of radon progeny exposure from mine air, was calculated for each individual as the product of the exposure time in working months (1 month = 170 hr) and average working level to estimate the cumulative radon exposure for each miner. Working level was defined as the measure of the energy released by airborne radon progeny that was measured based on counting the emissions of alpha particles in a representative volume of air [National Research Council (NRC) 1994]. One working level equals any combination of radon progeny in 1 L of air that results in the ultimate emission of 130,000 MeV of energy from alpha particles. The information used for WLM assessment was obtained from the mining history for each miner and from the accumulated mine measurement data collected by the National Institute for Occupational Safety and Health (NIOSH) (Archer et al. 2004; Lundin et al. 1971). WLM was calculated up to the time of cancer diagnosis for squamous cell carcinoma cases and for the entire uranium mining occupation for the controls.

*GENEVA GWAS of Lung Cancer and Smoking.* Lung cancer cases (*n* = 2,522) and controls (*n* = 2,725) drawn from the Environment and Genetics in Lung Cancer Etiology (EAGLE) study (1,816 cases and 1,984 controls) and the Prostate, Lung, Colon and Ovarian (PLCO) cancer screening trial (706 cases and 741 controls) comprised the GENEVA GWAS of Lung Cancer and Smoking (Landi et al. 2009), with genotype data obtained using Illumina HumanHap550v3.0 chips (dbGaP Study Accession number: phs000093.v2.p2; see Supplemental Material, Table S1). The EAGLE and PLCO studies enrolled participants from general populations in the Lombardy region of Italy and in 10 locations throughout the United States, respectively (see Supplemental Material, “Introduction for GENEVA GWAS of Lung Cancer and Smoking,” for a detailed introduction of these two studies); thus, participants in these studies would not be expected to have occupational exposure to radon or other radiation. Allele dosage for rs1800797 was extracted from imputation data that were obtained using European populations from the 1000 Genomes Project as the reference (1000 Genomes Project Consortium et al. 2012). This study was selected to assess whether the association with lung cancer that was observed for rs1800797 in miners can be generalized to populations with residential radon exposure because of the large sample size and available smoking history data. Stratification analysis by smoking status or tumor histology was conducted because a combined analysis of seven large-scale case–control studies conducted in North America identified a significant association between residential radon exposure and lung cancer risk in adenocarcinoma (Krewski et al. 2006). In addition, the role of residential radon exposure in the etiology of lung cancer may be more prominent in never smokers (Samet et al. 2009). The informed consent document signed by the PLCO study participants allows use of these data by investigators for discovery and hypothesis generation in the investigation of the genetic contributions to cancer and other adult diseases as well as for development of novel analytical approaches for GWASs. Use of the EAGLE data set is limited to scientific genetic research related to the etiology, molecular basis, and outcome of lung disease and smoking. Thus, IRB approval was not required for the use of these deidentified data for genetic association analysis in the present study.

*SNP selection and genotyping.* All common SNPs (minor allele frequency > 0.05) within the *IL6* locus (chr7: 22721293–22776691, genome build 36; see Supplemental Material, Figure S1) were predicted for their functional potential using SNP Web Info (Xu and Taylor 2009). Four SNPs (rs12700386, rs2069827, rs1800797, and rs2069840) in promoters not in high linkage disequilibrium (LD) (*r* ^2^ < 0.22) were suggested to affect the binding of transcription factors as indicated by the fact that MATCH^TM^ (Kel et al. 2003) predicted a transcriptional factor binding site with one allele but not with the other or the difference in the matrix similarity scores or core similarity scores between the two alleles was ≥ 0.2, and thus were selected for genotyping using the TaqMan genotyping assay (Life Technologies). The rs1800797 *A* allele is in almost perfect LD with the rs1800795 *C* allele (*r* ^2^ = 0.97). Participants with missing genotype data for more than two SNPs (5 squamous cell carcinoma cases and 23 controls) were excluded from the present analysis. Thus, 242 cases and 336 controls were eventually included in the genetic association analysis.

*Real-time polymerase chain reaction.* RNA was isolated from low-passage (≤ 2) primary human bronchial epithelial cell (BEC) cultures (*n* = 85) and human skin fibroblast lines (*n* = 6) that were established from cells obtained by bronchoscopy from current or former smokers examined at pulmonary clinics (Leng et al. 2012) or were obtained from infant foreskin at University of New Mexico Hospital, respectively. BEC cultures established from unaffected sites were used for this experiment for patients with subsequent lung cancer diagnosis. TaqMan real-time polymerase chain reaction (qPCR) was performed to quantify *IL6* gene expression in cDNA using the delta threshold cycle method with β-actin as the endogenous control.

*Resequencing of the* IL-6 *promoter.* Twenty-one alleles of the *IL6* promoter (690 bps, chr7:22732707–22733396) that contained 9 rs1800797 *A* alleles and 12 rs1800797 *G* alleles were successfully amplified using primers listed in Supplemental Material, Table S2, from 13 human BEC cultures that were heterozygous for rs1800797 and were directionally cloned into the pGL2-basic luciferase reporter vector (Promega) upstream of the luciferase coding sequence using *Mlu*I and *Bgl*II cloning sites according to the manufacturer’s instructions. Bidirectional Sanger sequencing was performed to confirm the haplotype alleles that contained four SNPs: rs1800797, rs1800796, rs36215814, and rs1800795.

*Luciferase reporter assay.* pGL2 constructs generated using the method described above that contained two common haplotype alleles (*A*-*G*-*A*8*T*12-*C* and *G*-*G*-*A*10*T*11-*G*) of the *IL6* promoter were sequence verified for transfection experiments. Transfection was performed in a human embryonic kidney cell line and a human lung fibroblast line (HEK293 and HFL1, respectively; both obtained directly from the American Type Culture Collection) using TransIT-2020 transfection reagent (Mirus Bio LLC) and in a normal human bronchial epithelial cell line (HBEC2) immortalized by insertion of the telomerase catalytic subunit and cyclin-dependent kinase 4 (obtained from Shay and Minna, Southwestern Medical Center, Dallas, TX) using a Neon electroporation system (Life Technologies). Cells were harvested 48 hr after transfection, and reporter activity was measured using the Dual Luciferase Assay System (Promega). Control experiments were conducted in the same manner as described above, except the *IL6* promoter constructs were replaced with promoterless or *SV40* promoter constructs. The constructs carrying two major haplotype alleles (*A*-*G*-*A*8*T*12-*C* and *G*-*G*-*A*10*T*11-*G*) in the *IL6* promoter generated 5–30 times higher reporter activity relative to the promoterless construct in the three cell lines tested (not shown), suggesting that *IL6* has a robust promoter.

**Figure 1:**
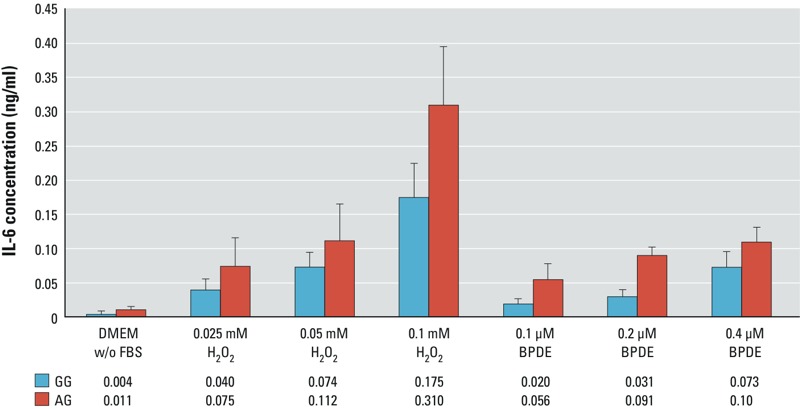
Promoter single nucleotide polymorphisms (SNPs) affect IL-6 secretion in fibroblast cells treated with H_2_O_2_ and benzo[*a*]pyrene diolepoxide (BPDE). Abbreviations: DMEM, Dulbecco’s modified essential medium; FBS, fetal bovine serum. Six fibroblast cell lines, three wild homozygotes (*GG*), and three heterozygotes (*AG*) for rs1800797 were treated with H_2_O_2_ and BPDE. The height of the bar is the average concentration of IL-6 in culture medium that is also expressed as the number under the figure. The error bar is the standard deviation from three independent experiments. A generalized linear model was used to assess the effects of H_2_O_2_ or BPDE treatment and rs1800797 genotype on IL-6 concentration detected in the medium. A strong IL-6 induction was identified for both carcinogens (*p*-values < 0.0001). The slope for the induction of IL-6 secretion by H_2_O_2_ or BPDE treatment is 74% and 39% greater in *AG* lines than *GG* lines (*p*-values = 0.017 for H_2_O_2_ and 0.13 for BPDE).

*Benzo[*a*]pyrene diolepoxide– and hydrogen peroxide–induced IL-6 secretion in fibroblast lines.* Lung fibroblasts appear to be a major source of IL-6 in the microenvironment of normal lungs based on the findings of greater basal and tobacco carcinogen–induced secretion of IL-6 in human lung fibroblasts than in lung epithelial cells (Chen et al. 2012). A dose-dependent induction of IL-6 was observed in lung fibroblasts treated with benzo[*a*]pyrene diolepoxide (BPDE) (Chen et al. 2012), and this response was replicated in skin fibroblasts (Figure 1). Thus, owing to the lack of lung fibroblasts with varied genotypes, the effect of *IL6* variants on IL-6 induction by lung carcinogen treatment was assessed in skin fibroblasts that were wild homozygous (*GG*, *n* = 3) and heterozygous (*AG*, *n* = 3) for rs1800797. Low-passage skin fibroblast lines (*n* = 6) were maintained in Dulbecco’s modified essential medium (DMEM) with 10% fetal bovine serum (FBS). Prior to treatment with BPDE or hydrogen peroxide (H_2_O_2_), cells were washed twice with phosphate-buffered saline (PBS) to remove any residual FBS in the medium. Cells were then cultured in DMEM without FBS and treated with BPDE or H_2_O_2_ at varied concentrations for 1 hr. The medium with carcinogens was then replaced with fresh medium without FBS. The level of IL-6 in the medium was measured 24 hr later using an enzyme-linked immunosorbent assay (ELISA) kit (eBioscience).

*Statistical analysis.* Logistic regression was used to estimate odds ratios (ORs) and 95% confidence intervals (CIs) for associations between case–control status and each SNP using an additive inheritance model that evaluated the contribution of each allele to cancer risk. Models were adjusted for cumulative radon exposure (WLM), which was modeled using one indicator variable for ≥ 0.895 kWLM and one indicator variable for missing WLM (*n* = 27), with < 0.895 kWLM as the common reference exposure, and for smoking pack-years (≥ 35 vs. < 35) and age at sputum collection. The missing indicator variable for WLM was included to maximize the number of observations included in the analysis.

The complete occupational histories for underground uranium mining were available for 162 miners with squamous cell carcinoma diagnosis, which allowed calculation of the mid-induction latency (MIL), defined as the time from the midpoint of the period of substantial exposure (an annual accumulative dose of ≥ 50.6 mSv) to squamous cell carcinoma diagnosis (Archer et al. 2004). All 162 miner cases achieved this level of exposure before the mid-1960s, when effective radiation control began to be implemented in underground uranium mines. The least-square means of MIL for miners with *GG*, *GA*, and *AA* genotypes of rs1800797 were calculated using a generalized linear model with adjustment for underground mining history and smoking history surveyed at cancer diagnosis. Cox proportional hazard models were used for 162 cases to estimate associations between *IL6* variants and the time to cancer diagnosis. Underground mining history (age at start of underground uranium mining, average WLM per month, and total years of underground mining) and smoking history (age at start of cigarette smoking, average number of cigarettes smoked per day, total years of cigarette smoking, and number of years after quitting smoking) surveyed at squamous cell carcinoma diagnosis were included in the model for covariate adjustment.

A generalized linear model was used to assess the association between rs1800797 and *IL6* expression in 85 primary human BECs and between subsequent lung cancer diagnosis and *IL6* expression in a subset of primary human BECs with adjustment for rs1800797 genotype coded as 0, 1, and 2 for *GG*, *GA*, and *AA*, respectively (*n* = 78). A likehood ratio test implemented in Merlin (Abecasis et al. 2002) was applied to test the association between rs1800797 and steady-state *IL6* expression (GI_10834983-S) in 79 Epstein-Barr virus (EBV)–transformed lymphoblastoid cell lines from CEU families (Utah residents with ancestry from northern and western Europe) in the HapMap project (Abecasis et al. 2002; Holm et al. 2010). The effects of rs1800797 and dose of carcinogen exposure on IL-6 secretion in human skin fibroblast lines (*n* = 6) were assessed using generalized linear models. The comparison of slopes for the dose–response curves by rs1800797 genotype was conducted using the likelihood ratio test by including an interaction term for genotype and carcinogen treatment in the generalized linear models. A two-sided *p*-value of 0.05 was used to define a significant association. Statistical analyses were conducted using SAS 9.2 (SAS Institute Inc.).

## Results

*Demographics of the miners.* A total of 242 cases and 336 controls were eventually included in the genetic association analysis (Table 1). Cases were older and had more pack-years than controls at the time of the latest sputum collection. Half of the cases died within 6 months of diagnosis. Cases were an average of 6 years younger at death than controls. All study participants were male and of non-Hispanic white (NHW) ethnicity.

**Table 1:** Characteristics of squamous cell carcinoma cases and controls.

Variable	Case	Control	*p*-Value
*n*	242	336
Age at lung cancer diagnosis (years, mean ± SD)	60.3 ± 10.4	—
Survival after diagnosis [years, median (Q1–Q3)]	0.6 (0.1–2.3)	—
Age at death (years, mean ± SD)	62.6 ± 10.6	69.2 ± 12.5	2.8 × 10^–1^^0^^*a*^
Sex (male percent)	100	100
Ethnicity (non-Hispanic white %)	100	100
WLM [kWLMs,^*b*^ median (Q1–Q3)]	1.0 (0.5–2.1)	0.8 (0.4–1.5)	0.001^*c*^
< 0.895 (%)	41.1	54.0	0.055^*d*^
≥ 0.895 (%)	48.6	45.4
Missing (%)	10.4	0.6
Mid-induction latency [years, median (Q1–Q3)]^*e*^	19 (12.0–26.5)
WLM, working level month. ^***a***^Student’s *t*-test. ^***b***^Twenty-seven miners had missing cumulative WLM estimates. WLM, a time-integrated measure, was calculated as the product of time in working months (1 month = 170 hr) and working levels to estimate the cumulative exposure to radon daughter radiation for each miner. ^***c***^Wilcoxon rank-sum test. ^***d***^Chi-square test for differences between cases and controls. The missing group was not included in this test. ^***e***^Squamous cell carcinoma cases (*n *= 162) with complete information for latency and genotypes. Mid-induction latency defined as time from midpoint of the period of substantial exposure (an annual accumulative dose of ≥ 50.6 mSv) to squamous cell carcinoma diagnosis (Archer et al. 2004).			

*Association between* IL6 *promoter SNPs and squamous cell carcinoma in miners.* Miners with cumulative radon exposure levels ≥ 0.895 kWLM had a statistically significant greater risk for squamous cell carcinoma than miners with < 0.895 kWLM (OR = 1.51, 95% CI: 1.05, 2.18, *p* = 0.026 with adjustment for rs1800797 genotype, pack-years, and age at sputum collection), consistent with radon progeny exposure as a risk factor for squamous cell carcinoma in miners. More cases had missing WLM estimates than controls (OR = 18.6, 95% CI: 4.2, 82.8, *p* = 0.00012). The variant allele *A* of rs1800797 was a statistically significant risk factor for squamous cell carcinoma in former uranium miners (OR = 1.36, 95% CI: 1.05, 1.75, *p* = 0.018; Table 2). A sensitivity analysis of miners with nonmissing WLM estimates did not change the genetic associations observed for *IL6* promoter SNPs (OR = 1.34, 95% CI: 1.04, 1.73, *p* = 0.023).

**Table 2:** Association between IL-6 promoter single nucleotide polymorphisms (SNPs) and squamous cell carcinoma in miners (odds ratios based on adjusted logistic regression models of 242 cases and 336 controls) and hazard ratios for the time from the onset of high exposure to diagnosis among 162 cases.

SNP	Allele^*a*^	Case–control study of squamous cell carcinoma^*b*^			Mid-induction latency^*c*^	
		Case^*d*^	Control^*d*^	OR (95% CI)^*e*^	Average (years)^*f*^	Hazard ratio^*e*^
rs12700386	*C*/*G*	0.66/0.29/0.05	0.65/0.31/0.04	0.94 (0.69, 1.30)	19.3/20.1/18.4	0.81 (0.59, 1.11)
rs2069827	*G*/*T*	0.84/0.15/0.01	0.83/0.14/0.04	0.84 (0.56, 1.26)	19.5/19.9/13.8	1.30 (0.86, 1.95)
rs1800797	*G*/*A*	0.30/0.50/0.21	0.39/0.45/0.16	1.36 (1.05, 1.75)	20.7/19.1/18.8	1.57 (1.22, 2.01)
rs2069840	*C*/*G*	0.45/0.45/0.10	0.44/0.37/0.19	0.77 (0.60, 1.01)	20.1/19.3/17.5	0.95 (0.71, 1.28)
Abbreviations: CI, confidence interval; OR, odds ratio. ^***a***^All forward strand RefSNP alleles. Alleles underlined were minor allele and test allele in the logistic regression. ^***b***^Adjustment for cumulative working level month (WLM) estimate, pack-years and age at sputum collection was included in logistic regression models. Analysis was conducted in 242 lung squamous cell carcinoma cases and 336 controls. ^***c***^Adjustment for underground mining history and smoking history surveyed at squamous cell carcinoma diagnosis was included in Cox regression models. Analysis was conducted in 162 cases owing to unavailability of the complete record of the occupational history for underground uranium mining. ^***d***^Forward slashes separate the frequencies of wild homozygotes, heterozygotes, and variant homozygotes for each individual SNP. ^***e***^OR or hazard ratio was calculated for the association between genotype of each *IL6* variant and risk for squamous cell carcinoma or latency, respectively. Each variant was coded as 0, 1, or 2 for wild homozygote, heterozygote, or variant homozygote. ^***f***^Forward slashes separate the average mid-induction latency for participants carrying wild homozygotes, heterozygotes, and variant homozygotes for each individual SNP. Mid-induction latency was defined as time from midpoint of the period of substantial exposure (an annual accumulative dose of ≥ 50.6 mSv) to squamous cell carcinoma diagnosis (Archer et al. 2004).						

*Association between* IL6 *promoter SNPs and latency in miners with squamous cell carcinoma.* The median of latency (19 years) in the 162 miners with squamous cell carcinoma was comparable to that seen for a large group of underground uranium miners (18.9 years, *n* = 505) from the United States who were either current smokers or had quit smoking < 10 years when diagnosed with lung cancer (Archer et al. 2004). The least-square means of MIL calculated using a generalized linear model with adjustment for underground mining history and smoking history surveyed at cancer diagnosis were 20.7 (standard error, 0.68), 19.5 (0.50), and 18.0 (0.73) for miners with *GG*, *GA*, and *AA* genotypes of rs1800797, respectively (*p* = 0.0075). Consistent with being a risk allele for squamous cell carcinoma, each copy of the *A* allele of rs1800797 was associated with a hazard ratio (HR) of 1.57 (95% CI: 1.22, 2.01, *p* = 0.00037) for latency (Table 2).

*Association between rs1800797 and risk for lung cancer in the GENEVA dataset.* The GWAS of 2,522 lung cancer cases and 2,725 controls identified an increased risk for lung cancer associated with the rs1800797 *A* allele (OR = 1.10, 95% CI: 1.01, 1.20, *p* = 0.04; Table 3). Associations between rs1800797 and lung cancer varied significantly by smoking status, with OR = 1.41 (95% CI: 1.05, 1.91) for each *A* allele in never smokers (interaction *p*-value = 0.05 for the difference from current smokers); OR = 1.08 (95% CI: 0.95, 1.24) for each *A* allele in former smokers (interaction *p*-value = 0.03 for the difference from current smokers); and OR 1.06 (95% CI: 0.92, 1.22) in current smokers (Table 3). Associations with each rs1800797 *A* allele also varied by histologic subtype, with a significant positive association with adenocarcinoma (OR = 1.16; 95% CI:1.04, 1.31; *p* = 0.009) compared with squamous cell carcinoma (OR = 1.08; 95% CI: 0.93, 1.25; *p* = 0.3) (Table 3).

**Table 3:** Association between rs1800797 and risk for lung cancer in the GENEVA dataset.

Variable	Control (*n*)^*a*^	Case (*n*)^*a*^	OR (95% CI)	*p*-Value
Overall	0.67 ± 0.67 (2,725)	0.70 ± 0.67 (2,522)	1.10 (1.01, 1.20)	0.045^*b*^
Smoking status^*c*^
Never smokers	0.60 ± 0.63 (633)	0.70 ± 0.64 (138)	1.41 (1.05, 1.91)	0.024^*d*^
Former smokers	0.67 ± 0.68 (1,125)	0.72 ± 0.69 (1,204)	1.08 (0.95, 1.24)	0.24^*d*^
Current smokers	0.70 ± 0.68 (967)	0.67 ± 0.66 (1,180)	1.06 (0.92, 1.22)	0.43^*d*^
Histology
Adenocarcinoma	0.67 ± 0.67 (2,725)	0.72 ± 0.69 (986)	1.16 (1.04, 1.31)	0.0091^*d*^
Squamous cell carcinoma	0.67 ± 0.67 (2,725)	0.69 ± 0.67 (582)	1.08 (0.93, 1.25)	0.33^*d*^
Small cell	0.67 ± 0.67 (2,725)	0.62 ± 0.65 (256)	0.90 (0.73, 1.11)	0.32^*d*^
Others	0.67 ± 0.67 (2,725)	0.70 ± 0.67 (698)	1.04 (0.91, 1.19)	0.59^*d*^
Abbreviations: CI, confidence interval; GENEVA, Gene Environment Association Studies; OR, odds ratio. ^***a***^*A* allele dosage for rs1800797 presented as mean ± standard deviation. ^***b***^Adjustment for age, sex, smoking status, pack-years, and cohort was included in logistic regression models. ^***c***^The interaction terms between never versus current smokers and rs1800797 and between former versus current smokers and rs1800797 were included together in the logistic regression with statistical significance identified for both interaction terms (*p*-values = 0.050 and 0.029, respectively). ^***d***^Adjustment for age, sex, pack-years, and cohort was included in logistic regression models.				

*Association between rs1800797 and* IL6 *expression.* The risk allele of rs1800797 was associated with increased *IL6* expression in an allelic dose-dependent manner assessed in 85 primary human BECs from current and former smokers (*p* = 0.009) and 79 lymphoblastic cell lines from the HapMap CEU population (*p* = 0.034) (Table 4). Interestingly, human BECs collected from patients with a subsequent lung cancer diagnosis (*n* = 56) had significantly higher *IL6* expression than those without (*n* = 22) (0.0029 ± 0.0034 vs. 0.0013 ± 0.0014, *p* = 0.027 with adjustment for the rs1800797 genotype).

**Table 4:** Association between rs1800797 and *IL6* expression in primary human bronchial epithelial cells and lymphoblastic cell lines.

Tissue^*a*^	rs1800797	*n*	*IL6* expression (mean ± SD)	*p*-Value
Human bronchial epithelial cells				0.009^*b*^
	*GG*	42	0.0016 ± 0.0019
	*GA*	32	0.0031 ± 0.0036
	*AA*	11	0.0041 ± 0.0038
Lymphoblastic cell lines				0.034^*c*^
	*GG*	18	6.18 ± 0.33
	*GA*	43	6.19 ± 0.33
	*AA*	18	6.36 ± 0.37
^***a***^Primary human bronchial epithelial cell (BEC) cultures (*n *= 85) were established from cells obtained by bronchoscopy from current or former smokers examined at the pulmonary clinic of University of New Mexico Hospital. BEC cultures established from unaffected sites were used for this experiment for patients with subsequent lung cancer diagnosis. Epstein-Barr virus–transformed lymphoblastoid cell lines (*n *= 79) from CEU families (Utah residents with ancestry from northern/western Europe) were established by the HapMap project. ^***b***^Generalized linear model with adjustment for lung cancer diagnosis status. rs1800797 genotype was coded as 0, 1, or 2 for *GG*, *GA*, or *AA* genotype, respectively. *IL6* expression was expressed as relative quantification with β-actin as the endogenous control. ^***c***^Likelihood ratio test implemented in MERLIN was used to evaluate the association between rs1800797 and *IL6* expression (GI_10834983-S). The family structure and sex were included in the models for covariate adjustment.				

*Haplotype alleles in the* IL6 *promoter. AGC* and *GGG* are two major haplotype alleles in the *IL6* promoter that contain rs1800797, rs1800796, and rs1800795 and have cumulative allele frequency > 0.95 in European populations from the 1000 Genomes Project (1000 Genomes Project Consortium et al. 2012). However, the phasing status is unclear between rs36215814 as an *A*n*T*n polymorphism located between rs1800796 and rs1800795 and the other three SNPs. Cloned sequencing of 21 *IL6* promoter alleles amplified from 13 self-identified non-Hispanic white study participants who were heterozygous for rs1800797 identified eight different compositions of the *A*n*T*n polymorphism with *A*8*T*12 enriched in the *AGC* allele and *A*10*T*11 enriched in the *GGG* allele (see Supplemental Material, Table S2). According to the allele frequencies for *AGC* (0.5) and *GGG* (0.45) in the European populations (1000 Genomes Project Consortium et al. 2012), the estimated frequencies for haplotype alleles *A*-*G*-*A*8*T*12-*C* and *G*-*G*-*A*10*T*11-*G* are 0.5 and 0.15, respectively.

**Figure 2:**
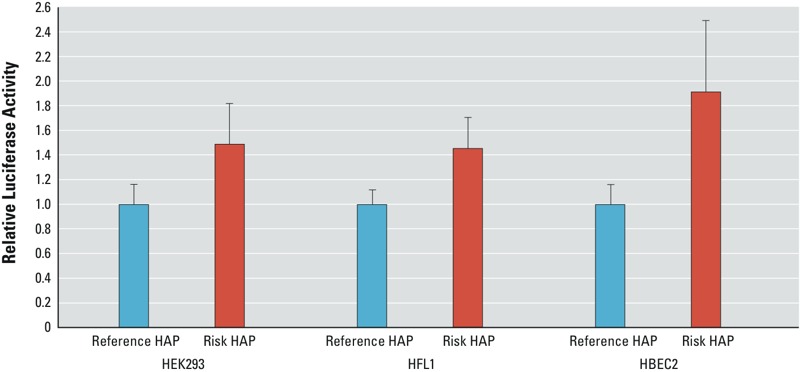
*IL6* promoter activity by haplotype alleles. Luciferase reporter construct containing haplotype allele *A*-*G*-*A*8*T*12-*C* (Risk HAP) that carried variant alleles for rs1800797 (*A*) and rs1800795 (*C*) had significantly higher reporter activity than was observed for *G*-*G*-*A*10*T*11-*G* (Reference HAP) in HEK293 (*p*-value = 3.42 × 10^–6^), HFL1 (*p*-value = 1.86 × 10^–5^), and HBEC2 (*p*-value = 6.2 × 10^–4^). The height of the bar is the average luciferase activity standardized by the levels observed for *G*-*G*-*A*10*T*11-*G* set to one. The error bar is the standard deviation.

IL6 *promoter activity by haplotype alleles.* The reporter assay showed that haplotype allele *A*-*G*-*A*8*T*12-*C* carrying the risk allele of rs1800797 had 45–92% increased promoter activity compared with *G*-*G*-*A*10*T*11-*G* in HFL1, HEK293, and HBEC2 cell lines (*p*-values < 0.00062; Figure 2).

IL6 *promoter SNPs and the effects of H_2_O_2_ and BPDE on IL-6 secretion in human fibroblasts.* Six skin fibroblast lines (three *GG*s and three *AG*s for rs1800797 as described in “Methods”) were used to assess whether the rs1800797 genotype could modify IL-6 secretion as the response to DNA damage indicative of radon or tobacco carcinogen exposure. *IL6* mRNA expression was 10 times higher in *AG* lines than in *GG* lines (0.0021 ± 0.0011 vs. 0.00020 ± 0.00009, respectively; *p* = 0.061, data not shown). Treating cells with H_2_O_2_ and BPDE creates oxidative damage and BPDE-DNA adducts that mimic the damage induced by radon and by the tobacco carcinogen benzo[*a*]pyrene, respectively (Narayanan et al. 1997; Tellez et al. 2011). The effects of the rs1800797 genotype (coded as 0 for *GG* and 1 for *AG*) and the dose of carcinogen (concentration in medium) on IL-6 secretion in human skin fibroblast lines (*n* = 6) were assessed using generalized linear models. A dose-dependent induction of IL-6 secretion was seen in skin fibroblasts treated with H_2_O_2_ and BPDE (*p*-values < 0.0001; Figure 1). *AG* lines had consistently higher levels of IL-6 secretion than *GG* lines (*p*-values < 0.015) with both treatments. Moreover, the slopes for the induction of IL-6 secretion by increasing concentrations of H_2_O_2_ and BPDE were 74% and 39% greater, respectively, in *AG* lines than in *GG* lines [2.97 (0.44) vs. 1.71 (0.20), *p* = 0.017 for H_2_O_2_ and 0.24 (0.04) vs. 0.17 (0.02), *p* = 0.13 for BPDE], indicating a stronger induction kinetic for IL-6 secretion in *AG* lines. Furthermore, the strong correlation between *IL6* mRNA expression and protein secretion (Pearson correlation coefficient = 0.78) suggested that the IL-6 secretion induced by carcinogen treatment stems from gene transcription.

## Discussion

In this study, we comprehensively evaluated the association between four *IL6* promoter variants predicted by *in silico* analyses to affect binding of transcription factors and lung squamous cell carcinoma in former uranium miners with high levels of radon exposure. We found that rs1800797 was significantly associated with increased odds for squamous cell carcinoma and shortened latency for development of squamous cell carcinoma. In particular, rs1800797 was associated with increased basal expression and induced secretion of IL-6 by fibroblasts in response to DNA damage *in vitro*, supporting a role for IL-6 in the association between rs1800797 and squamous cell carcinoma in the former uranium miners. The association between rs1800797 and squamous cell carcinoma in the former uranium miners was somewhat consistent with the association between rs1800797 and all lung cancers among never smokers in the GENEVA GWAS of Lung Cancer and Smoking study population, although the association was stronger for adenocarcinoma than for squamous cell carcinoma when evaluated by case subtype. Radon exposures were not assessed in GENEVA participants, but radon is a potential cause of lung cancer among never smokers. This finding reinforces the importance of considering the levels of environmental exposures when studying the association between *IL6* promoter variants and environmental disease phenotype (Cole et al. 2010; Ljungman et al. 2009b). In addition, a stratified analysis by tumor histology identified a significant association between rs1800797 and lung adenocarcinoma, a result consistent with the finding that > 73% of lung cancer patients who were never smokers were diagnosed with adenocarcinoma in the GENEVA dataset.

The association between the *IL6* promoter variants and increased risk for lung cancer was strongly supported by functional studies. We found that rs1800797 was significantly associated with increased *IL6* mRNA expression in primary HBECs, lymphoblastic cells, and fibroblasts, which may at least partially stem from the increased gene transcription assessed using the luciferase reporter assay. Our cloning strategy recapitulated the physical linear allelic combination for the four common SNPs at this 690-bp promoter region for the two common haplotype alleles studied (*A*-*G*-*A*8*T*12-*C* and *G*-*G*-*A*10*T*11-*G*) and allows for the detection of potential combined function of the four individual SNPs on gene transcription, a key factor in detecting the difference in basal expression of *IL6* (Terry et al. 2000). Other studies that compared luciferase activities for two promoter constructs that differed by only one base (rs1800795, *G*/*C*) with one allele not naturally existing did not see an increase in basal transcription associated with the rs1800795 *C* allele (Cole et al. 2010; Fishman et al. 1998; Kristiansen et al. 2003).

Compelling evidence suggests that IL-6 signaling in the tumor microenvironment is an essential factor promoting tumor cell proliferation, survival, and metastasis (Fisher et al. 2014). Consistent with this supposition, our previous studies support a paracrine-dominant mechanism for IL-6 signaling mediated by lung fibroblasts that increases the risk of malignant transformation in human bronchial epithelial cells (Chen et al. 2012). In contrast, inhibition of IL-6 secretion in lung fibroblasts greatly reduced transformation of bronchial epithelial cells exposed chronically to tobacco carcinogens (Chen et al. 2012). In the present study, we observed a strong induction of IL-6 secretion in fibroblasts with exposure to H_2_O_2_ and BPDE. Because treatment of cells with H_2_O_2_ and BPDE creates oxidative damage and BPDE-DNA adducts that mimic the damage induced by radon and the tobacco carcinogen benzo[*a*]pyrene (Narayanan et al. 1997; Tellez et al. 2011), respectively, these findings further implicate the involvement of IL-6 in promoting the development of lung squamous cell carcinoma in uranium miners. Of great importance, assessment of the effects of the rs1800797 genotype on the slopes for IL-6 induction by H_2_O_2_ or BPDE treatments identified greater induction of IL-6 secretion in rs1800797 *AG* lines than in *GG* lines. Thus, the elevated levels of basal and carcinogen-induced IL-6 secretion observed in fibroblasts carrying IL-6 variants could lead to a microenvironment that may favor clonal expansion and progression of lung premalignant field defects. This microenvironment would in turn contribute to an increased risk for lung cancer in populations with exposure to radon and/or to cigarette smoke.

Controls were selected from uranium miners who had no lung cancer diagnosis during their entire lives; thus, our study design was optimal for a genetic association study because no misclassification for a control becoming a case was possible. However, caution should be taken in the interpretation of the associations between radon exposure, smoking history, and risk for lung cancer. WLM was calculated up to cancer diagnosis for cancer cases and for the entire uranium-mining occupation for the controls. Smoking history was obtained at the time of sputum collection with the assumption that heavy smokers (≥ 35 pack-years) would maintain a heavy smoking status for their entire lifetime. It is unknown whether smoking behavior changed after sputum was collected. Thus, radon and cigarette smoke exposures were not estimated for controls at the point when controls had person-time contributions proportional to those of cases at cancer diagnosis. A nested case–control study design may be more appropriate to address the temporal relationship between radon exposure, smoking history, and risk for lung cancer.

## Conclusions

Our findings suggest that sequence variants in the *IL6* promoter modulate gene transcription and responses to environmental carcinogens by lung fibroblasts. In particular, *in vitro* evidence that the rs1800797 variant modulated *IL6* expression supports a role for IL-6 in pathogenic mechanisms associated with squamous cell lung cancer in uranium miners and with lung cancer in never smokers.

## Supplemental Material

(436 KB) PDF
