# Supplementary material for: Radon Exposure, IL-6 Promoter Variants, and Lung Squamous Cell Carcinoma in Former Uranium Miners
Source: Environ Health Perspect. 2015 Sep 15;124(4):445–51. doi: 10.1289/ehp.1409437 (PMC4829998; doi:10.1289/ehp.1409437)
Supplement: (436 KB) PDF [file ehp.1409437.s001.acco.pdf]

*Environ Health Perspect*
DOI: 10.1289/ehp.1409437

**Note to Readers:** *EHP* strives to ensure that all journal content is accessible to all readers. However, some figures and Supplemental Material published in *EHP* articles may not conform to 508 standards due to the complexity of the information being presented. If you need assistance accessing journal content, please contact ehp508@niehs.nih.gov. Our staff will work with you to assess and meet your accessibility needs within 3 working days.

# Supplemental Material

# Radon Exposure, IL-6 Promoter Variants, and Lung Squamous Cell Carcinoma in Former Uranium Miners

Shuguang Leng, Cynthia L. Thomas, Amanda M. Snider, Maria A. Picchi, Wenshu Chen, Derall G. Willis, Teara G. Carr, Jacek Krzeminski, Dhimant Desai, Amin Shantu, Yong Lin, Marty R. Jacobson, and Steven A. Belinsky

## Table of Contents

**Introduction for GENEVA GWAS of Lung Cancer and Smoking study**

**Figure S1.** The LD map for common SNPs (MAF > 0.05) within a 56kb region surrounding IL-6 using HapMap CEU data was shown in 1A. The boundaries were determined as the ends of two haplotype blocks. The coordinates were according to genome build 36. The common haplotype alleles (frequency > 0.05) within each block are shown in 1B. The multiallelic D' for the common haplotype alleles between the two blocks is 0.90, suggesting the level of recombination between the two blocks <10%. The rs number for the SNPs in 1B can be identified from 1A by finding the corresponding numbers on top of the haplotype allele in 1B and the LD map in 1A.

**Table S1.** Demographics for GENEVA GWAS dataset

**Table S2.** Summary of haplotype alleles identified using clone sequencing

**Introduction for GENEVA GWAS of Lung Cancer and Smoking study (http://www.ncbi.nlm.nih.gov/projects/gap/cgi-bin/study.cgi?study_id=phs000093.v2.p2)**

The Environment and Genetics in Lung Cancer Etiology Study (EAGLE, 1816 cases and 1984 controls) and the Prostate, Lung, Colon and Ovary Study (PLCO, 706 cases and 741 controls) Cancer Screening Trial comprised the GENEVA GWAS of Lung Cancer and Smoking study (Landi et al. 2009)

The Environment and Genetics in Lung Cancer Etiology Study (EAGLE) is a large population-based case-control study designed and conducted to investigate the genetic and environmental determinants of lung cancer and smoking persistence using an integrative approach that allows combined analysis of genetic, environmental, clinical, and behavioral data. The study setting, the Lombardy region of Italy, is served by a network of modern hospitals, medical schools, and a regional health service. Within the Lombardy region, the catchment's area includes 5 cities and 216 surrounding municipalities, encompassing, in the selected age range, over 1.3 million people. Cases were identified through a group of hospitals which include catchment of greater than 80% of the lung cancer cases in a defined geographic area in the Lombardy region of Italy including Milan, with a first diagnosis of lung cancer between April 22, 2002 and February 28, 2005. The case group consisted of ~2,000 newly incident lung cancer cases, born in Italy, of Italian nationality, with official residence in one of 216 selected municipalities, male and female, 35 to 79 years old, collected before or after surgery and prior to chemotherapy or radiation therapy. All histologic types and stages of lung cancer are represented. Cases were consecutively collected in the departments of thoracic surgery, general surgery, general medicine, and oncology of the participating hospitals. Subjects in intensive care units, or with cardiac, hepatic, renal, or CNS failure, or with uncompensated schizophrenia,

psychosis, or inability to speak were excluded. The control group consisted of ~2,000 gender and age- and region- matched subjects selected through the Regional Health Services Database. The healthy controls were randomly selected from the same residential area of the lung cancer cases. Other exclusion criteria are the same as for the case group. All study participants provided written informed consent.

The Prostate, Lung, Colorectal and Ovarian Cancer Screening Trial (PLCO) is a randomized screening study with a cohort design. The purpose of the PLCO Trial is to determine whether certain cancer screening tests reduce deaths from these cancers of the prostate, lung, colorectum, and ovaries. Together, these four cancers account for about 60% of all diagnosed cancers in the United States. Enrollment opened in 1993 and ended in 2001, with over 155,000 men and women participating at 10 locations throughout the United States. Patients are stratified by participating center, gender, and age (55-59 vs 60-64 vs 65-69 vs 70-74) and randomized to one of two treatment arms. In the Control arm, patients receive standard medical care. In the Screened arm, all patients undergo sigmoidoscopic examination and chest x-ray; men also undergo DRE (Digital rectal exam) and PSA (Prostate-specific antigen) testing and women undergo a transvaginal ultrasound and CA-125 (Cancer Antigen-125) test. A scheduling and tracking procedure is implemented to ensure regular attendance at repeat screens for subjects screened negative or for those who are designated suspicious or positive at screening but for whom subsequent diagnostic procedures do not reveal prostate, lung, colorectal, or ovarian cancer (follow-up diagnostic procedures are through their own medical care environment). Patients diagnosed via a screening test with cancer of the prostate, lung, colorectum, or ovary are referred for treatment in accordance with current accepted practice for appropriate stage of disease, patient age, and medical condition; DRE (men only), transvaginal ultrasound (women

only), and chest x-ray are repeated annually for 3 years. Patients who have never smoked do not receive a third chest x-ray. A Periodic Survey of Health questionnaire is mailed to each participant annually for 13 years to identify all prevalent and incident cancers of the prostate, lung, colorectum, and ovary as well as all deaths that occur among both screened and control subjects during the trial.

**Figure S1.** The LD map for common SNPs (MAF > 0.05) within a 56kb region surrounding IL-6 using HapMap CEU data was shown in 1A. The boundaries were determined as the ends of two haplotype blocks. The coordinates were according to genome build 36. The common haplotype alleles (frequency > 0.05) within each block are shown in 1B. The multiallelic D' for the common haplotype alleles between the two blocks is 0.90, suggesting the level of recombination between the two blocks <10%. The rs number for the SNPs in 1B can be identified from 1A by finding the corresponding numbers on top of the haplotype allele in 1B and the LD map in 1A.

Figure S1A.

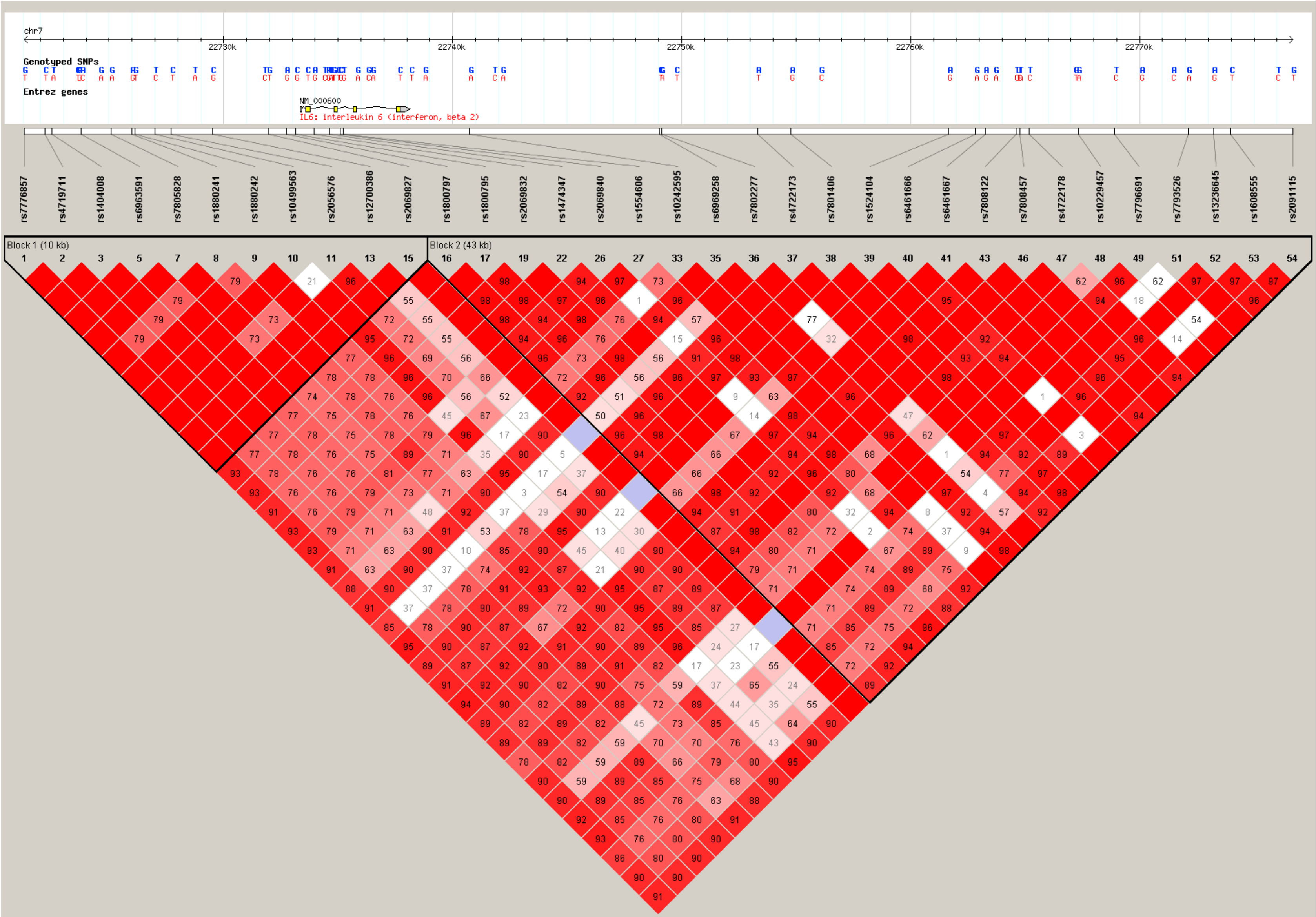
chr7
22730k
22740k
22750k
22760k
22770k
Genotyped SNPs
Entrez genes
NM_000600
IL6: interleukin 6 (interferon, beta 2)
rs7776857
rs4719711
rs1404008
rs6963591
rs7805828
rs1880241
rs1880242
rs10499563
rs2056576
rs12700386
rs2069827
rs1800797
rs1800795
rs2069832
rs1474347
rs2069840
rs1554606
rs10242595
rs6969258
rs7802277
rs4722173
rs7801406
rs1524104
rs6461666
rs6461667
rs7808122
rs7808457
rs4722178
rs10229457
rs7796691
rs7793526
rs13236645
rs1608555
rs2091115
Block 1 (10 kb)
Block 2 (43 kb)

Figure S1B.

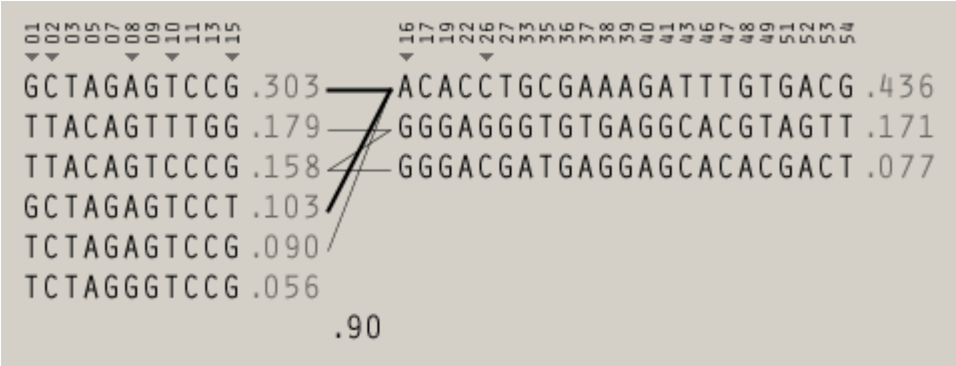
01 02 03 05 07 08 09 10 11 13 15
16 17 19 22 26 27 33 35 36 37 38 39 40 41 43 46 47 48 49 51 52 53 54
GCTAGAGTCCG .303
TTACAGTTTGG .179
TTACAGTCCCG .158
GCTAGAGTCCT .103
TCTAGAGTCCG .090
TCTAGGGTCCG .056
ACACCTGCGAAAGATTTGTGACG .436
GGGAGGGTGTGAGGCACGTAGTT .171
GGGACGATGAGGAGCACACGACT .077
.90

**Table S1.** Demographics for GENEVA GWAS dataset [a]

| Variable | Control | Case | p-Value |
|---|---|---|---|
| N | 2725 | 2522 | |
| PLCO, % | 27.2 | 28.0 | 0.79 [b] |
| Age, % | | | |
| 59 or less | 24.5 | 20.2 | <0.0001 [b] |
| 60 – 64 | 21.6 | 19.1 | |
| 65 – 69 | 25.5 | 24.8 | |
| 70 – 74 | 18.5 | 21.8 | |
| 75 or higher | 9.9 | 14.1 | |
| Male gender, % | 71.8 | 74.7 | 0.02 [b] |
| NHW ethnicity, % | 100 | 100 | |
| Smoking status, % | | | |
| Never | 23.2 | 5.5 | <0.0001 [b] |
| Former | 41.3 | 47.7 | |
| Current | 35.5 | 46.8 | |
| Packyears, % | | | |
| 0 | 23.2 | 5.5 | < 0.0001 [b] |
| 0-15 | 25.6 | 8.5 | |
| 15-30 | 17.1 | 15.2 | |
| 30-40 | 13.3 | 18.5 | |
| 40-50 | 7.8 | 15.5 | |
| 50-60 | 5.0 | 12.8 | |
| 60-70 | 2.6 | 7.3 | |
| 70-80 | 1.9 | 4.9 | |
| > 80 | 3.5 | 11.9 | |
| Tumor stage, % | | | |
| I and II | | 34.5 | |
| III and IV | | 65.5 | |
| Histology, % | | | |
| Adenocarcinoma | | 39.1 | |
| Squamous cell carcinoma | | 23.1 | |
| Other NSCLC [c] | | 11.7 | |
| Small cell | | 10.2 | |
| Other lung cancer | | 6.8 | |
| Missing | | 9.2 | |
| Rs1800797, A allele dosage (mean ± SD) [e] | 0.67 ± 0.67 | 0.70 ± 0.67 | 0.06 [d] |

[a] EAGLE is a population-based, biologically intensive, case-control study from the Lombardy region of Italy including ~2000 newly diagnosed lung cancer cases and ~2000 age-, gender- and region- matched controls. PLCO Cancer Screening Trial contributes ~850 lung cancer cases and ~850 age- and gender- matched controls.

[b] $\chi^2$ test for differences between cases and controls.

[c] Other NSCLC included large cell lung cancer and other NSCLC.

[d] Two-sided Wilcoxon rank sum test

[e] A allele dosage is the estimate for number of A allele with the highest posterior probability.

**Table S2.** Summary of haplotype alleles identified using clone sequencing [a]

| rs1800797 | rs1800796 | rs36215814 | rs1800795 | Count |
|---|---|---|---|---|
| A | G | 8A12T | C | 6 |
| A | G | 8A10T | G | 1 |
| A | G | 8A12T | G | 1 |
| A | G | 10A11T | G | 1 |
| G | C | 10A10T | G | 4 |
| G | G | 8A12T | C | 1 |
| G | G | 9A11T | C | 1 |
| G | G | 10A11T | G | 2 |
| G | G | 9A10T | G | 1 |
| G | G | 9A11T | G | 1 |
| G | G | 10A12T | G | 1 |
| G | G | 9A12T | G | 1 |

[a] The primers sequences for cloning are AATGCACGCGTACCTGGAGACGCCTTGAA (forward) and ATCTGAGATCTCTGGAGGGGAGATAGAGCTTC (reverse). DNA samples were acquired from 13 human BEC cultures that were heterozygotes of rs1800797.
